# COVID-19 Vaccination Intent, Barriers and Facilitators in Healthcare Workers: Insights from a Cross-Sectional Study on 2500 Employees at LMU University Hospital in Munich, Germany

**DOI:** 10.3390/vaccines10081231

**Published:** 2022-07-31

**Authors:** Ana Zhelyazkova, Selina Kim, Matthias Klein, Stephan Prueckner, Sophia Horster, Philipp Kressirer, Alexander Choukér, Michaela Coenen, Kristina Adorjan

**Affiliations:** 1 Institute of Emergency Medicine and Management in Medicine, University Hospital, Ludwig Maximilian University of Munich, 80336 Munich, Germany; selina.kim@med.uni-muenchen.de (S.K.); stephan.prueckner@med.uni-muenchen.de (S.P.); 2 Department of Neurology, University Hospital Munich, Ludwig Maximilian University of Munich, 81377 Munich, Germany; matthias.klein@med.uni-muenchen.de; 3 Administrative Department, University Hospital Munich, Ludwig Maximilian University of Munich, 81377 Munich, Germany; sophia.horster@med.uni-muenchen.de; 4 Department of Communication and Media, University Hospital Munich Ludwig Maximilian University of Munich, 80336 Munich, Germany; philipp.kressirer@med.uni-muenchen.de; 5 Laboratory of Translational Research Stress and Immunity, Department of Anaesthesiology, University Hospital Munich, Ludwig Maximilian University of Munich, 81377 Munich, Germany; alexander.chouker@med.uni-muenchen.de; 6 Institute for Medical Information Processing, Biometry, and Epidemiology—IBE, Chair of Public Health and Health Services Research, Ludwig Maximilian University of Munich, 81377 Munich, Germany; coenen@ibe.med.uni-muenchen.de; 7 Pettenkofer School of Public Health, 81377 Munich, Germany; 8 Department of Psychiatry and Psychotherapy, University Hospital Munich, Ludwig Maximilian University of Munich, 80336 Munich, Germany; kristina.adorjan@med.uni-muenchen.de

**Keywords:** COVID-19, vaccination, vaccination hesitancy, healthcare workers, health belief model, vaccination, non-pharmaceutical interventions

## Abstract

Considering the role of healthcare workers (HCW) in promoting vaccine uptake and previously recorded hesitancy among HCW, we aim to examine the COVID-19 vaccination intent and status of HCW through a cross-sectional anonymous online survey at LMU University Hospital in Munich. Data collection was informed by the Health Belief Model (HBM) and focused on vaccination intent, status and on potential factors affecting the decision-making process. In total, 2555 employees completed the questionnaire. Our data showed that an approving attitude towards recommended vaccines and having received an influenza vaccine in the previous winter were strongly associated with COVID-19 vaccination intent. Further, a positive COVID-19 vaccination status was associated with a higher likelihood of approving the extension of the validity of non-pharmaceutical interventions at the workplace. Our HBM-analysis demonstrated strong associations between the perceived benefits and barriers and COVID-19 vaccination intent. Unchanged or low perceived susceptibility and severity were associated with refusal or indecisiveness. Our findings highlight the factors associated with the decision regarding a COVID-19 vaccine and indicate a pattern-like behavior in the acceptance of novel vaccines by HCW. These insights can help inform the communication aims of vaccination campaigns among HCW within similar organizational contexts or in future outbreaks.

## 1. Introduction

One of the top priorities in the World Health Organization’s strategic pandemic management has been defined as achieving a global COVID-19 vaccination coverage by the middle of 2022 [1]. In order to achieve this milestone, healthcare workers (HCW) take on a particularly important role due to their high risk of contracting and spreading an infectious disease in a nosocomial context as well as due to their essential function in healthcare services provision [2]. Furthermore, HCW are considered to be gatekeepers and trusted sources of information regarding vaccination among their patients as well as the general population [3,4]. However, diverging attitudes and intentions regarding immunizations can be observed among HCW, as well [4,5]. 

During the 2009/2010 pandemic influenza (pH1N1) outbreak, unexpectedly low levels of pH1N1 vaccination were reported among HCW worldwide [6]. Although the spread of SARS-CoV-2 presents a different pandemic context, fluctuations in the intent of HCW to receive a COVID-19 vaccine can be observed. The “All Corona Care” study conducted in 2020 (May to July) at LMU University hospital, one of the largest in Germany, asked participants prior to the authorization of any COVID-19 vaccine if they would be willing to get vaccinated if there were an efficient vaccine available with few side effects. Out of 7554 participants in the study, 58.2% were willing to get vaccinated [7]. Still, it remained to be explored if and how the vaccination intent in this HCW cohort would shift after the authorization of the first COVID-19 vaccines as well as which factors and aspects drive the decision-making process towards accepting, delaying or refusing a vaccination.

This study aims to examine the COVID-19 vaccination intent and vaccination status of HCW at one of the largest hospitals in Germany and to identify factors associated with the COVID-19 vaccination intent and vaccination status. The methodological framework of this study rests upon the Health Belief Model (HBM) as one of the most established theoretical concepts in health behaviour research and a preferred concept in the examination of the individuals’ acceptance and attitudes towards health promoting and disease preventing behaviours and measures [8,9]. The methods are further informed by the measurement recommendations of the SAGE Working Group on Vaccine Hesitancy in regard to vaccination intent [10,11].

Specifically, in this report, we aim to provide evidence on the topic by examining the following sets of hypotheses:

1. General attitude towards vaccines and COVID-19 vaccines;
2. Attitude towards other non-pharmaceutical interventions (NPIs) following a COVID-19 vaccination;
3. Factors associated with the intent to vaccinate (informed by the HBM).

## 2. Materials and Methods

We conducted a cross-sectional anonymous online survey targeted at all employees of LMU University Hospital in Munich between 25 February and 20 March 2021 to gather data regarding the intent to receive the COVID-19 vaccine as well as the factors associated with the decision. LimeSurvey Version 4.4.12+210308 was used for the design of the questionnaire. 

The survey was conducted as part of a prospective study to evaluate the vaccination process at the LMU University Hospital in the course of the COVID-19 pandemic (IMPF^LMU^). The study was reviewed and approved by the ethics committee of the medical faculty at LMU Munich, Germany (Project number: 21-0123). 

With 11,070 employees and 101 departments, the LMU University Hospital is the second largest university hospital in Germany [12]. The vaccination campaign against COVID-19 began in 27 December 2020 and proceeded until 17 July 2021, thus being among the first hospitals in Germany that set up a vaccination centre and a large-scale vaccination campaign for their employees [13]. At the point of the launch of the survey, approx. one third of the hospital’s employees had received at least one vaccine dose. The vaccination campaign was set up with several consecutive prioritisation phases, where hospital personnel with the highest COVID-19 infection risk were the first to receive a vaccination appointment (e.g., personnel of the emergency department, COVID-19 departments). Due to ministerial distribution algorithms, the LMU University hospital vaccination centre exclusively used the vaccine “Comirnaty” (BNT162b2) during this vaccination campaign, however, employees were not deterred from attending an appointment at a different vaccination centre (e.g., communal centres). Hospital employees vaccinated at a different vaccination centre were also able to participate in the survey. For the purposes of this study, we define vaccine hesitancy as a delay in acceptance or refusal of vaccines despite availability of vaccine services [11]. 

The questionnaire was developed based on the in-house evaluation needs as well as on a literature review. The questions were categorized in six sections: (1) general media consumption (two questions), (2) in-house media consumption (three questions), (3) in-house media consumption regarding COVID-19 vaccinations (three questions), (4) general attitude towards vaccines (three questions), (5) general attitudes towards COVID-19 vaccines (eight questions) and (6) socio-demographic data (seven questions). The design and selection of questions for sections 1, 4 and 5 were informed by the previous work of the WHO Strategic Advisory Group of Experts (SAGE) as well as by the implementation of the HBM in predicting health behaviours [8,9,10,11,14]. Previous studies on the acceptance of COVID-19 and other vaccines have showcased the suitability of HBM for exploring this type of research questions [15,16,17].

The questionnaire was designed in German and translated into English for the purposes of this publication.

The primary outcome of the survey was the intent to receive a vaccination against COVID-19 (section 5). The main question gathering information regarding the intent to vaccinate was formulated as “Are you going to get vaccinated against COVID-19?” and provided four options to respond: “Yes,” “No,” “Maybe,” and “I have already received one or both of the vaccination doses”. The latter option was necessary as the vaccination campaign had begun approximately two months (28 December 2020) prior to the rollout of the survey (25 February 2021). Participants who had selected “Yes” or “I have already received one or both of the vaccination doses” were forwarded to a multiple-choice question about their reasons for wanting to receive a COVID-19 vaccine. Participants who had selected “No” were asked, through a multiple-choice question, about their reasons for denying a COVID-19 vaccine. Participants who had indicated indecisiveness (“Maybe”) were presented with a multiple-choice question on the factors that could potentially serve as motivators for them to receive a COVID-19 vaccine.

Responses on the main question were used to build two variables: one indicating intent to vaccinate (containing the responses “Yes,” “No” and “Maybe”, excluding already vaccinated participants) and one indicating the vaccination status (dichotomous “vaccinated” and “not vaccinated”). The newly created variables were used for testing the presented hypotheses. Perceived susceptibility, severity, benefits and barriers were measured using a 5-point Likert scale. The Likert scale was subject to regrouping, since, for the majority of the items, the original scale did not provide a subgroup sample size large enough to execute the multinomial regression. Furthermore, the consistent regrouping of the 5-point Likert scale enhances the comparability of results.

Due to the absence of a universally agreed upon process-oriented methodology in HBM research, we opted for an individual examination of the associated constructs instead of parallel, serial or a moderated analysis [18]. Further, we executed the analyses in an itemized manner in order to provide insights into the specific aspects driving the association between HBM constructs and vaccination intent.

The survey (including information about the IMPF^LMU^ study) was disseminated through a designated intranet page as well as through the employee newsletter, available to all employees. Several reminders were sent between 25 February and 20 March 2021. All employees of the hospital were eligible to participate in the survey.

### Statistical Analysis

Descriptive analysis was performed for the sociodemographic data as well as the data on internal communication, general communication (partially) and on the responses regarding the reasoning for the vaccination decision. Potential confounders and effect modifiers (age, sex, occupational category, education and direct work with COVID-19 patients) were tested for significant associations with vaccination intent and status using a Pearson’s Chi-square test (Table 1). Only significant variables were included in the following analyses (α= 0.05).

All hypotheses were tested for both vaccination intent and status, where multinomial logistic regression models were used for vaccination intent (AIC, BIC) and binomial logistic regression models were applied to test the vaccination status (Cox and Snell R-Quadrat, Nagelkerkes R-Quadrat). The hypotheses on the HBM-based cues to action (H3) as well as on the attitude towards NPIs (H2) were examined with a multinomial logistic regression (AIC, BIC). One hypothesis on the utilization of media and perceived knowledgeability was tested with a generalized linear model (Pearson’s Chi-square Test). 

Due to the small subgroup size in some variables where the data were collected using a 5-point Likert scale, items on the lower end of the scale (coded “1” and “2”) as well as items on the upper end of the scale (coded “4” and “5”) were respectively collapsed, thus providing a variable with three response options.

Data processing and analyses were conducted with IBM SPSS Statistics Version 26.0.0.0. Depending on the model fit, the unadjusted models were preferred for interpretation. Unreported models are presented as tables in Supplement B.

## 3. Results

In total, 3590 of 11070 employees (32.4%) of LMU University hospital participated in the survey. Of those, 2555 completed the questionnaire in full. Only fully completed questionnaires were considered for further analysis. Table 1 shows the frequency and distribution of the socio-demographic and occupational characteristics of participants as well as the distribution of vaccination status and intent among respondents. Table 2 provides insight into the reasons of participants for their vaccination decision. The data show that protecting oneself and one’s close ones dominates arguments for receiving a COVID-19 vaccine, whereas uncertainty about the vaccines’ effectiveness and safety were leading causes for refusal and uncertainty. 

For vaccination intent, age and education showed weak positive associations (Table 1). For vaccination status, all tested variables showed a weak positive association except for the dichotomous variable for occupation, which demonstrated a strong negative association (Table 1). 

All variables with an association on vaccination intent or status were included in the adjusted multinomial and binomial logistic models, respectively. 

### 3.1. General Attitude towards Vaccines and Influenza Vaccine Uptake

We examined the association between the general attitude towards vaccines and the COVID-19 vaccination intent (Table 3). The data show a strong association between capacity of an individual’s opinion about generally receiving the recommended vaccinations and one’s intent to get vaccinated against COVID-19. Respondents who do not or only partially agree with the statement that everyone should receive the recommended vaccines had a significantly higher probability of refusing a COVID-19 vaccine. Equivalently, respondents who do not or only partially agree with the statement had a significantly higher probability of being undecided on whether or not to get vaccinated. 

In terms of dealing with negative comments (e.g., comments on ineffectiveness, harms) regarding vaccines in general as a predictor for COVID-19 vaccination intent, the data show only a limited effect. Regarding dealing with negative comments, people who do (often) deal with negative comments had a significantly higher probability of refusing a COVID-19 vaccine (Table 3). 

The influenza vaccine uptake in the winter of 2020/2021 was associated with COVID-19 vaccination intent. The data in the better fitted unadjusted model show that people who were vaccinated against influenza at the end of 2020 or beginning of 2021 have a significantly higher probability of accepting a COVID-19 vaccination. 

The results are similar for respondents who do not or only partially agree with the statement that everyone should receive the recommended vaccines, being significantly less likely to have already been vaccinated against COVID-19 (Table 4). Further, people who were vaccinated against influenza in the winter of 2020/2021 have a significantly higher probability of having already received a COVID-19 vaccine (Table 4).

### 3.2. Attitude towards Other Non-Pharmaceutical Interventions Following A COVID-19 Vaccination

We examined whether the vaccination status is associated with a certain attitude towards NPIs (e.g., mask mandate, visitor regulations) at the LMU University hospital (Table 5). HCW who agreed with preventative measures remaining until the end of 2021 were more likely to have already been vaccinated. However, HCW who did not agree or only partially agreed with extending the measures to 2022, as well, were less likely to have already been vaccinated against COVID-19. Further, HCW who did not agree with extending the offer for free PCR-testing at the hospital despite the progress of the vaccination campaign were more likely to not have been vaccinated.

### 3.3. Factors Associated with Vaccination Intent (Informed by the Health Belief Model)

#### 3.3.1. Perceived Susceptibility

We tested the perceived susceptibility to COVID-19 with five items assessing one’s perceived likelihood to get infected as well as one’s attitude change towards the likelihood of getting infected in a private or professional setting since the beginning of vaccination (α = 0.509) [14]. HCW who disagreed or rather disagreed with the statement that they were less worried about attracting COVID-19 in a professional setting compared to before the start of the vaccination campaign had a significantly higher likelihood of not intending to receive a COVID-19 vaccine (Table 6) or being still undecided on the matter. HCW who partially agree with the statement are significantly more likely to be undecided regarding a COVID-19 vaccine.

For vaccination status, HCW who disclosed to being less worried about getting infected in their professional or personal setting since the beginning of vaccination were more likely to have already received one or both vaccination doses (Table 7).

#### 3.3.2. Perceived Severity of Disease in Case of Attraction of COVID-19

For perceived severity, we tested two items (α = 0.817). Unlike perceived susceptibility, the items for perceived severity demonstrated only one borderline significance towards vaccination intent, where people who identify their risk of getting sick from COVID-19 as low or very low were significantly more likely to be undecided (Table 6).

In terms of vaccination status, the data showed that persons who define their risk of getting sick from COVID-19 as very low or low are more likely to have already been vaccinated (Table 7).

#### 3.3.3. Perceived Benefits

The perceived benefits were measured with one item assessing the individual’s conviction of the effectiveness of COVID-19 vaccines (Table 6). The data in the better fitting unadjusted model showed a strong significant effect of low or partial conviction of the effectiveness of COVID-19 vaccines on the vaccination intent. Further, persons who are not or only partially convinced of the effectiveness are significantly more likely to be undecided on getting a COVID-19 vaccine than those who are rather or completely convinced.

The results are also reflected in the better fitting model adjusted for age, sex, education and occupation for outcomes for vaccination status, where HCW who are not or are only partially convinced of the effectiveness of COVID-19 vaccines are less likely to have already received a dose (Table 7).

#### 3.3.4. Perceived Barriers

Perceived barriers were measured with two items (α = 0.845). The results demonstrate a strong association between perceived barriers and the vaccination intent (Table 6). Respondents who are not or only partially convinced of the safety of COVID-19 vaccines are significantly more likely to refuse a vaccine or undecided on whether or not to get vaccinated. 

We observed similar results for the effect of concerns regarding COVID-19 vaccines on the vaccination intent (Table 6). People who have any concerns regarding the COVID-19 vaccines are significantly more likely to refuse a vaccine. Similarly, those with concerns or partial concerns have a significantly higher likelihood of being undecided.

In terms of vaccination status, the results in the better fitted adjusted model showed an identical result with people uncertain or concerned regarding COVID-19 vaccines having a higher chance of not being vaccinated (Table 7).

#### 3.3.5. Cues to Action

We analysed the cues to action by examining the link between the COVID-19 vaccination intent and the utilization of media platforms and channels (external cues) as well as the perceived knowledgeability on the topic (internal cues).

##### Perceived Knowledgeability and COVID-19 Vaccination Intent

We examined how the individual’s perceived knowledgeability on COVID-19 vaccines affects the intention to receive one (Table 8). Due to the relatively even distribution of subgroups, we decided against the merger of items as opposed to the other analysed HBM constructs. There was a particularly strong association for disagreeing or completely disagreeing with the statement “I generally felt well informed about COVID-19 vaccines and their safety” and being more likely to not have intent or being undecided on receiving a COVID-19 vaccine.

##### Utilization of Certain Media Platforms or Channels and Perceived Knowledgeability

We examined how the utilization of different media platforms or channels (both private, state, official and other channels) affects one’s perception of knowledgeability regarding COVID-19 vaccines with a generalized linear model (Table 9). Not discussing the topic of vaccination with other people as well as not getting involved in personal conversations with family members, friends or acquaintances was linked to a likely increase in perceived knowledge about COVID-19 vaccines. Similarly, seeking information specifically on vaccines may increase one’s perception of knowledgeability. On the contrary, perceived knowledgeability may be reduced if one does not turn to the information resources provided by state or federal health authorities or does not discuss vaccinations with the vaccination doctor or with another medical professional.

##### Utilization of Certain Media Platforms or Channels and The COVID-19 Vaccination Intent

We conducted the same analysis for the COVID-19 vaccination intent using a multinomial logistic regression (Table 10). For media platforms, the model showed a strong association between not using public television channels and refusing a COVID-19 vaccine. Not using social media networks or personal conversations with other people as an information source was linked to a lower risk of denying COVID-19 vaccination. Regarding indecisiveness, not using daily newspapers and podcasts was linked to a higher probability whereas not conversing with others was associated with a lower likelihood of being undecided. 

Further, not utilizing scientific sources, information from international organizations and pharmaceutical companies was found to reduce the risk of COVID-19 vaccine refusal. In contrast, not utilizing the information sources provided by state or federal health authorities was linked to a higher likelihood of vaccine refusal. 

Supplement A provides insights into the demands and expectations of participants regarding the design and contents of vaccine-related information and messages. Furthermore, statistics on the utilization of internal communication and information services are provided.

## 4. Discussion

The presented study shows an in-depth analysis of COVID-19 vaccination intent and vaccination status of HCW in one of the largest university hospitals in Germany at the beginning of the vaccination campaign (25 February to 20 March 2021); in comparison to a survey conducted prior to the authorization of any COVID-19 vaccine, vaccination intent in our cohort had increased [7]. Our data show that a generally approving attitude towards recommended vaccines and having been vaccinated against influenza in the winter of 2020/2021 were strongly associated with COVID-19 vaccination intent. Further, HCW that had already received at least one vaccine dose were more likely to agree with extending NPIs until the end of 2021. However, HCW not yet vaccinated were more likely to disagree or only partially agree with continuing the NPIs (including free PCR-testing) in 2022. Our HBM-based analysis of the factors influencing the decision-making processes on COVID-19 vaccination demonstrated particularly strong associations between perceived benefits and barriers and the refusal or indecisiveness regarding reception of the vaccine. Unchanged or rather low perceived susceptibility and severity were associated with reluctance or indecisiveness. In the analysis of cues to action, the results showed that HCW who perceive themselves as ill-informed about COVID-19 vaccines and their safety are significantly more likely to refuse vaccination or to be undecided. Factors associated with an increase in perceived knowledgeability regarding COVID-19 vaccines were not conversing with others (e.g., family members, acquaintances) but rather seeking specific information on the topic. A reduction in the perceived knowledgeability was observed in cases where information provided by sources such as state or federal health authorities as well as by healthcare professionals was not utilized. Further, there was a significant association between not conversing with others on the topic and being less likely to refuse or be undecided on whether or not to get vaccinated, similar to the results for the effect of personal conversations on one’s perceived knowledgeability. Not using social media as an information channel was linked to a lower likelihood of COVID-19 vaccination refusal. 

The results of this study contribute to the existing body of evidence on the intention and reasoning behind a vaccination decision of HCW in a pandemic context beyond COVID-19 [15]. The COVID-19 vaccination intent and status among the examined HCW cohort after the beginning of the vaccination campaign in Germany amplifies the evidence outlined by similar cross-sectional self-administered surveys among HCW, as these were conducted primarily prior to, rather than after, the approval of any COVID-19 vaccine. Two surveys among healthcare personnel in university hospitals in Italy and France present an intent to receive a COVID-19 vaccine of over 75% of respondents [19,20]. In a nationwide disseminated questionnaire in Italy, the results indicated a slightly lower rate, with 67% of respondents intending to vaccinate against COVID-19 as soon as a vaccine was available, 27.7% feeling uncertain and 7.3% refusing a vaccine [21]. A similar percentage (28.4%) of reluctance towards COVID-19 vaccines was reported among French-speaking HCW in France, Belgium and Canada [22]. A rather inhomogeneous vaccination intent was reported by six surveys conducted among HCW in hospital settings outside of Europe, with COVID-19 vaccination acceptance rates ranging between 27.7% and 63.0% [23,24,25,26,27,28]. A more recent survey conducted in two Vietnamese general hospitals after the approval of several vaccines has shown a significant acceptance of COVID-19 vaccines of 76.10% [29]. The comparably high COVID-19 vaccination intent identified in our analysis might suggest a longitudinal shift in HCW COVID-19 vaccination intent after the authorization of the first vaccines. A similar longitudinal shift has been observed by two German-wide surveys on HCW COVID-19 vaccination, where the vaccination intent increased from 65% to 75% between December 2020 and February 2021 in one of the surveys [30] and from 83% in March and April 2021, a period in which the presented data were also collected, to 91% and 92% in the second and third wave, respectively [31,32,33]. 

### 4.1. General Attitude towards Vaccines

Further, participants of the KroCo study, a longitudinal survey on COVID-19 vaccination intent by the Robert Koch Institute, also placed their main arguments against a COVID-19 vaccine in the concerns regarding side effects or even long-term damage as well as uncertainty regarding the vaccine’s technology. The main reasons for receiving a COVID-19 vaccine were similarly related to protecting one’s health as well as their close ones [31,32,33]. Several international studies observed similar arguments for and against getting a COVID-19 vaccination, with the protection of oneself and close ones being a main driver for and concerns about the safety, efficacy and side effects of vaccines as reasons against it [20,21,26,28,34]. Similar paths of reasoning were also observed in regard to the pandemic H1N1 (pH1N1) vaccination during the 2009/2010 outbreak [35,36,37]. Similarly, safeguarding one’s health and the health of their loved ones were previously identified as the main driver for receiving any vaccine by HCW [38]. 

At the time of preparation of this manuscript, we could not identify other studies exploring the association between a generally approving attitude towards vaccines and a positive COVID-19 vaccination intent. However, several studies in an international context have also demonstrated a significant relationship between seasonal influenza vaccination uptake and COVID-19 vaccination intent, corresponding to our findings [20,21,24,26,39]. In a historical analogy, a seasonal influenza vaccination was found to be a common predictor for intending to a receiving a pH1N1 influenza vaccination [15,35,36,37].

### 4.2. Attitudes towards Non-pharmaceutical Interventions

At the moment of preparation of these results, we could not identify other studies that had explored the association between COVID-19 vaccination status and attitude towards pandemic-related NPI in HCW populations. Thus, the outcomes presented here provide a reference for future research on the association between attitudes towards COVID-19 pharmaceutical and non-pharmaceutical measures.

### 4.3. Health Belief Model Constructs

Our findings concerning the HBM factors, however, build upon previously published theoretical and empirical evidence [8,9,40]. Wong et al. observed a very strong association between the items for perceived benefits and a COVID-19 vaccination intent [17]. Perceived benefits and severity were also positively correlated in a population-based study by Wong et al., while the perceived barriers showed a strong negative association with COVID-19 vaccination intent [16]. Similarly, a HBM-based study among Vietnamese HCW reported strong associations for cues to action, perceived benefits and barriers (negative association), whereas the association for perceived susceptibility and severity was relatively weaker [29].

Beyond the COVID-19 vaccine, the perceived benefits as well as the cues to action were identified by Shahrabani et al. as main HBM drivers for seasonal influenza vaccination among HCW in Israel [41]. 

It is important to note that when exploring potential COVID-19 vaccine decision drivers outside of HBM, several studies identified the perceived individual risk of COVID-19 (often using a factor combing perceived susceptibility and severity) as a strong predictor for HCW for receiving a COVID-19 vaccine [20,21,24]. The systematic review by Ahmad et al. further highlights the distrust in a vaccine’s content, safety, efficacy and side-effects as factors associated with vaccine hesitancy among HCW [42]. As these studies were conducted without the inclusion of other HBM constructs, it is not possible to reflect on the other potentially related factors.

In order to reflect on the fast-paced information background of COVID-19 vaccination campaigns, we attempted an itemized analysis of potential cues to action. Our data support previously published evidence on the significant correlation between cues to action and vaccination intent. In the study by Huynh et al., the cues to action account for the strongest association with a COVID-19 vaccination intent, although no further detail on the specific cues is provided [29]. We found that not utilizing the information provided by state or federal health authorities or not discussing vaccinations with the vaccination doctor or with another medical professional reduces the perceived knowledgeability regarding COVID-19 vaccines, which in turn reduces the likelihood of a vaccination intent. These results build a valuable analogy to the cues to action associated with a COVID-19 vaccination intent among the general population [16]. Further, corresponding to our results on the negative association between social media utilization and vaccination intent, Di Gennaro et al. observed that Italian HCW who were primarily using Facebook as an information source were significantly more likely be hesitant regarding a COVID-19 vaccine [21]. The utilization of social media platforms and its effect on one’s motivation to adopt preventive measures, more particularly a vaccination, has been previously examined through the lens of risk perception. However, the results on how and why social media usage affects COVID-19 risk perception, especially the intent to receive a COVID-19 vaccine, vary strongly depending on the target group and setting [43,44,45]. 

### 4.4. Limitations

Several limiting factors need to be taken into account when interpreting the results of this study. As to the survey design and conduction, only approximately one third of employees filled out the questionnaire of IMPF^LMU^. Further, occupational groups who are working at various locations at the hospital (e.g., logistics, hospital hygiene, catering services) participated less in the study. Due to the rapid rollout of the vaccination campaign at LMU University Hospital, the presented survey could not be launched before the beginning of vaccination. Consequently, a large proportion of the target population had already been vaccinated once when the survey was launched. This disrupted the initial timeline and lead to the addition of the fourth response option (“I have already received one or both of the vaccination doses”) to the question on vaccination intent. Further, changes in attitude may have occurred following the beginning of the vaccination campaign or after being vaccinated. Although the majority of the participants noted that information in the German language is sufficient, it is quite possible that the linguistic diversity of the hospital’s personnel was not well reflected among the study participants. Recent studies indicate that language barriers as well as ethnical and cultural differences significantly contribute to vaccine hesitancy [39,46].

Concerning results, the differences in subgroup sizes pose a challenge for the interpretation of the results. In addition, we cannot exclude the impact of social desirability bias as well as of central tendency bias on the responses of participants [47,48]. 

HBM-based analyses rest upon the psychosocial assumption of health being considered of high priority by the targeted population [49]. Although the results of this study do indicate a strong prioritization of one’s personal health as a facilitator for receiving a COVID-19 vaccine, further health and non-health related factors that may also influence the decision-making process but go beyond the scope of HBM should be considered in future research attempts. Relevant health-related factors in this sense include the health and well-being of persons in one’s professional (e.g., patients) and private network (e.g., family). Furthermore, non-health related factors represent a potential confounding aspect in HBM-based analyses. Additional aspects that could not be taken into consideration due to the cross-sectional design of this study are the potential change of attitude towards COVID-19 vaccines throughout the vaccination campaign, and the COVID-19 vaccination mandate for HCW adopted on 10 December 2021. Especially since the recommendations of the European Medicines Agency (EMA) as well as of Germany’s Standing Committee on Immunization (STIKO) underwent several updates in the first half of 2021, changes in attitude towards specific vaccines or COVID-19 vaccines in general are possible [50,51]. A further analysis into the cues to action would have been possible with a more detailed section on the utilization of information platforms and channels, as there are quantitatively and qualitatively diverse possibilities for employing information sources when actively or passively seeking information. This limitation is particularly valid in regard to social media utilization in terms of misinformation and infodemic management [49,52].

It should be noted that the presented study did not consider the possibility of a COVID-19 vaccination mandate for HCW and the therewith-associated labor and economic factors. The respective legislation was adopted on 10 December 2021 and binds a working contract in any healthcare institution to a complete COVID-19 vaccination as of 16 March 2022 [53].

## 5. Conclusions

Our findings provide insights into the vaccination intent and status of COVID-19 vaccines among HCW as well as on the reasons and factors affecting these. Our results can serve as guidance for the design of vaccination campaigns among HCW in similar organizational contexts as well as for the management of future epidemic or pandemic outbreaks. Further, the pronounced evidential comparisons between the vaccination intent and attitudes of HCW during the H1N1 and the COVID-19 pandemic indicate the existence of a pattern-oriented behaviour beyond contextual parameters. These indications would call for a holistic approach towards improving and accelerating the adoption of novel pharmaceutical measures (i.e., vaccines) by HCW through preventively addressing the here outlined determinants, barriers and modifiers of vaccination intent. 

Appropriately, our study contributes towards the development of a framework for health promotion communication targeted at HCW by identifying the specific aspects of HBM factors that could be addressed most efficiently. Further, the operationalization of HBM in this study caters to the empirical evidence for the application of the model in a healthcare setting within a pandemic context, particularly by presenting an in-depth perspective on the parameters and mechanism of impact of cues to action. 

## Figures and Tables

**Table 1:** Socio-demographic and occupational characteristics of participants.

	*n*	%	Coefficient *p-*Value	
Age *			Intent to Vaccinate	Vaccination Status
<29 years	487	19.1	0.130 0.000	0.081 0.005
30–39 years	604	23.6		
40–59 years	523	20.5		
50–69 years	683	26.7		
>60 years	239	9.4		
No answer	19	0.7		
Sex **			0.048 0.193	0.073 0.001
Female	1807	70.7		
Male	739	28.9		
Other	9	0.4		
**Education**			0.106 0.019	0.203 <0.001
Secondary/Elementary school	40	1.6		
Middle school	331	13.0		
High school/technical diploma	439	17.2		
Vocational training	497	19.5		
Academic degree (Bachelor)	193	7.6		
Academic degree (Master/Diploma)	420	16.4		
Academic degree (Doctorate or higher)	574	22.5		
Other training	60	2.3		
No diploma	1	0.0		
Occupation ***			0.036 0.426	−0.458 <0.001
Medical staff	1478	48.7		
Non-medical staff	1120	51.3		
**Work with COVID-19 patients**			0.051 175	0.257 <0.001
Yes	446	17.5		
Mean number of weeks **** = 19.27 (SD = 19.75, 1–60 weeks)				
No	2109	82.5		
**Vaccination status**				
Vaccinated	1235	48.3		
Not vaccinated	1320	51.7		
**Intent to receive a COVID-19 vaccine (not vaccinated)**				
Yes	1104	83.6		
No	82	6.2		
Maybe	134	10.2		
All (not vaccinated)	1320			
**All**	**2555**			

* Age group distribution at LMU University Hospital: <29 years = 22.9%, 30–39 years = 29.1%, 40–59 years = 18.8 %, 50–69 years = 20.9%, >60 years = 8.4%. ** Sex distribution at LMU University Hospital: Female = 66.3%, Male = 33.7%. *** Occupational distribution at LMU University Hospital: Medical staff = 45.4%, non-medical staff = 54.6%. **** The question was only available to fill out by participants who had selected “yes” to having had worked at a designated COVID-19 unit or with COVID-19 patients.

**Table 2:** Frequencies of the reasons for the respective decision on COVID-19 vaccine.

What are your main reasons for willing to receive a COVID-19 vaccine? *	***n***	**%**
To protect others (family, colleagues, patients)	2210	94.5%
To protect myself	2171	92.8%
I want to contribute to maintaining public health and achieving collective immunity	1839	78.6%
I am worried for my family and relatives	1523	65.1%
To participate in social activities again (restaurant visits, concerts etc.)	1428	61.1%
So I can travel again	1370	58.6%
I am fully convinced of the effectiveness and safety of COVID-19 vaccines	1245	53.2%
To lead with example at the hospital	1047	44.8%
I am afraid of getting seriously ill from COVID-19	851	36.4%
I am afraid of getting infected with COVID-19	835	35.7%
I work with COVID-19 patients	662	28.3%
I am not fully convinced by the effectiveness and safety of COVID-19 vaccines but I see those as the lesser of two evils	496	21.2%
I identify as a risk patient	407	17.4%
Due to societal expectations	107	4.6%
As to not be identified as an “antivaxxer”	34	1.5%
I work with very vulnerable patients	10	0.4%
What are the reasons for which you do not (yet) wish to receive a COVID-19 vaccine? **	***n***	**%**
I am afraid of the long-term (yet unknown) reactions to the vaccines	69	87.3%
I am not convinced of the safety and effectiveness of COVID-19 vaccines	67	84.8%
I have concerns due to the fast-tracked process of development	62	78.5%
I am still lacking evidence on the effectiveness and safety of COVID-19 vaccines	53	67.1%
I am lacking trust in the mechanism of mRNA vaccines	49	62.0%
I am lacking trust in the health institutions, pharma companies or the media	40	50.6%
I do not belong to a vulnerable group	31	39.2%
I am afraid of short-term reactions to the vaccines	25	31.6%
I am not prepared to get vaccinated in order to protect others	21	26.6%
I have no contact with COVID-19 patients	21	26.6%
I think the restrictions regarding hygiene (e.g., mask mandate) are enough	21	26.6%
It is unlikely for me to get ill from COVID-19	19	24.1%
I generally do not get vaccinated	13	16.5%
I’ve already had COVID-19 and did not perceive it as so bad	7	8.9%
I’ve already had COVID-19 and am hence immune	4	5.1%
Due to health reasons (incl. pregnancy)	3	3.8%
Due to cultural or religious reasons	2	2.5%
I currently have no time for a vaccine	1	1.3%
What could positively influence your willingness to receive a COVID-19 vaccine? ***	***n***	**%**
More evidence on the long-term effects of COVID-19 vaccines	109	82.6%
More scientific evidence on the safety of COVID-19 vaccines	87	65.9%
More scientific evidence on the effectiveness of COVID-19 vaccines	85	64.4%
More time between the market authorization and myself receiving the vaccine—I prefer to wait a little longer.	74	56.1%
A longer process of vaccine development	61	46.2%
An exhaustive explanation about the different mechanisms of COVID-19 vaccines	52	39.4%
More general information about COVID-19 vaccines (e.g., in media)	41	31.1%
My family and friends getting vaccinated and going through the process well	36	27.3%
Personal conversations with an expert	33	25.0%
Personal conversations with already vaccinated colleagues	31	23.5%
High incidence and mortality rates in my area	18	13.6%
Participation in vaccine trials	17	12.9%
Delay due to health reasons incl. pregnancy	5	3.8%

* This was a filtered question available only to those who had replied with “yes” or “I have already received one or both of the vaccination doses” to the previous question (“Are you going to receive a COVID-19 vaccine?”); *n* = 2339. ** This was a filtered question available only to those who had replied with “no” to the previous question (“Are you going to receive a COVID-19 vaccine?”); *n* = 82. *** This was a filtered question available only to those who had replied with “maybe” to the previous question (“Are you going to receive a COVID-19 vaccine?”); *n* = 134.

**Table 3:** Multinomial logistic regression of attitudes towards vaccinations associated with intent to receive a COVID-19 vaccine.

	Vaccination Intent				
“I Think It’s Important that Everyone Receives the Recommended Vaccinations.” *	Yes (ref.)	No		Maybe	
	*n*	*n* RR	95% CI	*n* RR	95% CI
Disagree/rather disagree	13	65 529.500	223.704–1253.308	32 50.130	24.840–101.169
Partly agree	32	7 23.166	8.288–64.753	50 31.821	18.846–53.728
“When you hear a negative comment about vaccine(s), do you:…..?” **	**Yes (ref.)**	**No**		**Maybe**	
	***n***	***n*** **RR**	**95% CI**	***n*** **RR**	**95% CI**
“Ask for the opinion(s) of those in your private environment”—no	862	60 0.685	0.392–1.194	89 0.486	0.319–0.740
“Get the opinion of a doctor or healthcare professional”—no	799	65 1.610	0.890–2.912	100 1.281	0.824–1.992
“Check the correctness of the statements through media reports”—no	328	30 1.421	0.741–2.725	43 0.997	0.606–1.638
“I do not (often) deal with negative comments”—no	865	73 2.393	1.041–5.499	111 1.111	0.638–1.935
“No answer”—no	1038	69 0.524	0.211–1.301	120 0.480	0.219–1.054
“I engage with the person expressing the negative comment”—no ***	1097	82		1134	
“Did you get vaccinated against influenza in 2020/21 season?” ****	**Yes (ref.)**	**No**		**Maybe**	
	***n***	***n*** **RR**	**95% CI**	***n*** **RR**	**95% CI**
“Yes”	665	13 0.124	0.068–0.228	29 0.182	0.119–0.280
“No” (ref.)	439	69		105	
All (not yet vaccinated)	**1104**	**82**		**134**	

* AIC = 39.633, BIC = 70.746 (unadjusted model); Reference category. Agree/rather agree; ** Multiple choice question, AIC =151.188 BIC = 161.558; Reference category in each item is the answer “yes” to executing the given action; *** Too few cases to allow for analysis; **** AIC = 29.799 BIC = 50.541.

**Table 4:** Binomial logistic regression of attitudes towards vaccinations associated with negative COVID-19 vaccination status.

“To What Extent Do You Agree with the following Statement? ”	Vaccination Status (Not Vaccinated)			
“I find it important for everyone to receive the recommended vaccinations”*	***n***	**OR**		**95% CI**
Disagree / rather disagree	110	0.138	.	0.080–0.237
Partly agree	89	0.577	.	0.385–0.865
“When you hear a negative comment about vaccine(s), do you…..” **	***n***	**OR**		**95% CI**
“Ask for the opinion(s) of those in your private environment”—no	1011	1.134	.	0.903–1.424
“Get the opinion of a doctor or healthcare professional”—no	964	0.893	.	0.721–1.105
“Check the correctness of the statements through media reports”—no	401	1.218	.	0.953–1.557
“I do not (often) deal with negative comments”—no	1049	0.893	.	0.689–1.158
“No answer”—no	1227	2.558	.	1.597–4.096
“I engage with the person expressing the negative comment”—no***	1313	−		−
All	**1320**			

* Cox and Snell R-Quadrat= 0.248; Nagelkerkes R-Quadrat = 0.331 (adjusted model for age, sex, education, occupation); Reference category; Agree/rather agree; ** Multiple choice question, Cox and Snell R-Quadrat = 0.234; Nagelkerkes R-Quadrat = 0.312; Reference category in each item is the answer “yes” to executing the given action (*** too few cases to allow for analysis).

**Table 5:** Binomial logistic regression of negative COVID-19 vaccination status associated with the attitudes towards other implemented non-pharmaceutical interventions.

“In General, Regarding the COVID-19 Vaccination Campaign, It Is Important for Me...”*	Vaccination Status (Not Vaccinated)			
**“...that the current measures at LMU University Hospital (e.g., mask mandate) remain valid until the end of 2021”**	***n***	**OR**		**95% CI**
Disagree	90	0.739	.	0.441–1.238
Rather disagree	85	0.845	.	0.522–1.365
Partly agree	235	1.104	.	0.809–1.506
Rather agree	347	1.302	.	1.009–1.681
**“...that the current measures at LMU University Hospital (e.g., mask mandate) remain valid in 2022 as well”**	***n***	**OR**		**95% CI**
Disagree	210	0.723	.	0.479–1.092
Rather disagree	216	0.634	.	0.441–0.912
Partly agree	439	0.715	.	0.533–0.958
Rather agree	228	0.833	.	0.608–1.140
**“...that testing at the LMU University Hospital should remain broadly available regardless of the vaccination campaign”**	***n***	**OR**		**95% CI**
Disagree	34	0.339	.	0.145–0.748
Rather disagree	23	0.583	.	0.273–1.245
Partly agree	76	1.007	.	0.654–1.550
Rather agree	361	0.925	.	0.654–1.550
All	1320			

* Cox and Snell R-Quadrat = 0.237; Nagelkerkes R-Quadrat = 0.316 (adjusted model for age, sex, education, occupation); the distribution of answers allowed for testing without merging any categories; Reference category in each item is the answer “Agree”.

**Table 6:** Multinomial logistic regression models with the Health Belief Model factors associated with intent to receive a COVID-19 vaccine.

Perceived Susceptibility Is a Predictor for Getting a COVID-19 Vaccine *	Vaccination Intent				
**α = 0.509 AIC =703.718, BIC = 714.088**	**Yes (ref.)**	**No**		**Maybe**	
**“How do you rate the following aspects from your personal point of view?”**	***n***	***n*** **RR**	**95% CI**	***n*** **RR**	**95% CI**
**“In regard to the spread of COVID-19 the likelihood that I myself be will infected is...”**					
Very low/Low	337	51 0.989	0.378–2.589	58 1.498	0.691–3.247
Medium	571	21 0.498	0.194–1.278	62 0.954	0.474–1.918
**“Since the vaccination campaign started, I’ve been more afraid of getting infected in my private environment than before or I’ve been more afraid for my loved ones.”**	***n***	***n*** **RR**	**95% CI**	***n*** **RR**	**95% CI**
Disagree/Rather disagree	892	76 0.862	0.290–2.560	106 0.736	0.334–1.625
Partly agree	152	2 1.007	0.239–4.250	20 0.918	0.362–2.326
**“Since the vaccination campaign started, I’ve been less afraid of getting infected in my private environment than before or I’ve been less more afraid for my loved ones.”**	***n***	***n*** **RR**	**95% CI**	***n*** **RR**	**95% CI**
Disagree/Rather disagree	571	70 2.155	0.894–5.196	90 1.905	0.947–3.833
Partly agree	255	2 0.456	0.122–1.699	31 1.909	0.899–4.057
**“Since the vaccination campaign started, I’ve been less afraid of getting infected in my professional environment than before.”**	***n***	***n*** **RR**	**95% CI**	***n*** **RR**	**95% CI**
Disagree/Rather disagree	575	71 3.094	1.180–8.114	93 3.231	1.527–6.839
Partly agree	248	3 0.595	0.205–2.479	30 2.283	1.051–4.961
**“Since the vaccination campaign started, I’ve been more afraid of getting infected in my professional environment than before.”**	***n***	***n*** **RR**	**95% CI**	***n*** **RR**	**95% CI**
Disagree/Rather disagree	925	78 6.007	1.909–18903	109 2.411	0.998–5.826
Partly agree	124	2 1.542	0.500–4.755	18 2.165	0.961–4.879
**Perceived severity is a predictor for a getting a COVID-19 vaccine**	**Yes (ref.)**	**No**		**Maybe**	
**α = 0.817 AIC = 82.230 BIC = 134.084**					
**“How do you rate the following aspects from your personal point of view?”**	***n***	***n*** **RR**	**95% CI**	***n*** **RR**	**95% CI**
**“In regard to the spread of COVID-19 the probability of me getting sick from COVID-19 is...”**					
Very low/Low	370	60 2.114	0.805–5.551	59 2.262	1.006–5.082
Medium	562	16 0.497	0.183–1.353	65 1.706	0.798–3.647
**“In regard to the spread of COVID-19 the probability of me getting seriously ill from COVID-19 is...”**	***n***	***n*** **RR**	**95% CI**	***n*** **RR**	**95% CI**
Very low/Low	654	72 7.874	0.952–65.149	91 1.538	0.581–4.070
Medium	342	9 3.981	0.464–34.146	37 1.446	0.546–3.830
**Perceived benefits are a predictor for a getting a COVID-19 vaccine**	**Yes (ref.)**	**No**		**Maybe**	
**AIC = 40.631 BIC= 71.743**			**95% CI**		**95% CI**
**“I am completely convinced of the effectiveness of the COVID-19 vaccines”**	*n*	*n* RR		*n* (RR; p-value)	
Disagree/Rather disagree	17	63 485.471	194.154–1213.891	46 72.979	37.977–140.241
Partly agree	170	12 9.247	3.589–23.824	54 8.567	5.412–13.561
**Perceived barriers are a predictor for a getting a COVID-19 vaccine**	**Yes (ref.)**	**No**		**Maybe**	
**α = 0.845 AIC = 93.445 BIC = 145.299**	***n***	***n*** **RR**	**95% CI**	***n*** **RR**	**95% CI**
**“I am completely convinced of the safety of the COVID-19 vaccines”**					
Disagree/Rather disagree	33	71 116.829	28.676–475.969	59 20.484	9.584–43.781
Partly agree	215	8 5.423	1.230–23903	57 5.938	3.115–11.322
**“I have no concerns regarding the COVID-19 vaccines”**					
Disagree/Rather disagree	93	73 10.264	2.916–36133	81 7.890	3.924–15.866
Partly agree	215	5 10.264)	0.348–6.924	36 2.744	1.366–5.513
**All**	**1104**	**82**			**134**

* Reference category in each item is the highest answer on the merged Likert scale (“Rather agree/Agree” or “High / Very high”); adjusted model for age, sex, education, occupation.

**Table 7:** Binomial logistic regression models with the Health Belief Model factors associated with intent to receive a COVID-19 vaccine.

Perceived Susceptibility Is a Predictor for Getting a COVID-19 Vaccine ^1,^*	Vaccination Status (Not Vaccinated)			
**“How do you rate the following aspects from your personal point of view?”**	***n***	**OR**		**95% CI**
**“In regard to the spread of COVID-19 the likelihood that I myself be will infected is...”**				
Very low/Low	446	0.644	.	0.430–0.965
Medium	654	0.920	.	0.654–1.295
**“Since the vaccination campaign started, I’ve been more afraid of getting infected in my private environment than before or I’ve been more afraid for my loved ones.”**				
Disagree/Rather disagree	1074	1.484	.	0.915–2.406
Partly agree	174	1.134	.	0.640–2.007
**“Since the vaccination campaign started, I’ve been less afraid of getting infected in my private environment than before or I’ve been less afraid for my loved ones.”**				
Disagree/Rather disagree	731	0.432	.	0.323–0.577
Partly agree	288	0.670	.	0.497–0.902
**“Since the vaccination campaign started, I’ve been less afraid of getting infected in my professional environment than before.”**				
Disagree/Rather disagree	739	0.249	.	0.187–0.332
Partly agree	281	0.525	.	0.395–0.697
**“Since the vaccination campaign started, I’ve been more afraid of getting infected in my professional environment than before.”**				
Disagree/Rather disagree	489	1.818	.	1.184–2.791
Partly agree	643	1.011	.	0.692–1.477
Perceived severity is a predictor for a getting a COVID-19 vaccine **	**Vaccination status (not vaccinated)**			
**“In regard to the spread of COVID-19 the probability of me getting sick from COVID-19 is...”**	***n***	**OR**		**95% CI**
Very low/Low	489	1.567	.	1.103–2.226
Medium	643	1.039	.	0.754–1.433
**“In regard to the spread of COVID-19 the probability of me getting seriously ill from COVID-19 is...”**				
Very low/Low	817	0.848	.	0.556–1.293
Medium	388	0.700	.	0.463–1.058
Perceived benefits are a predictor for a getting a COVID-19 vaccine ***	**Vaccination status (not vaccinated)**			
**“I am completely convinced of the effectiveness of the COVID-19 vaccines”**	***n***	**OR**		**95% CI**
Disagree/Rather disagree	126	0.061		0.032–0.118
Partly agree	236	0.554		0.428–0.718
Perceived barriers are a predictor for a getting a COVID-19 vaccine ****	**Vaccination status (not vaccinated)**			
**“I am completely convinced of the safety of the COVID-19 vaccines”**	***n***	**OR**		**95% CI**
Disagree/Rather disagree	163	0.189		0.107–0.331
Partly agree	280	0.704		0.528–0.939
**“I have no concerns regarding the COVID-vaccines”**				
Disagree/Rather disagree	247	0.436		0.296–0.642
Partly agree	256	0.739		0.555–0.985
All	**1320**			

^1^ Reference category in each item is the highest answer on the merged Likert scale (“Rather agree/Agree” or “High/Very high”). * Cox and Snell R-Quadrat = 0.334; Nagelkerkes R-Quadrat = 0.445 ** Cox and Snell R-Quadrat = 0.236; Nagelkerkes R-Quadrat = 0.314 *** Cox & Snell R-Quadrat = 0.264; Nagelkerkes R-Quadrat = 0.352 **** Cox and Snell R-Quadrat = 0.270; Nagelkerkes R-Quadrat = 0.360; unadjusted models.

**Table 8:** Multinomial logistic regression for the perceived knowledgeability associated with intent to receive a COVID-19 vaccine.

Perceived Knowledgability Is a Predictor of Intent to Receive a COVID-19 Vaccine *	Yes (ref.)	No		Maybe	
“I generally felt well informed about COVID-19 vaccines and their safety”	*n*	*n* RR	95% CI	*n* RR	95% CI
Disagree	30	24 25.900	10.690–62.752	22 21.104	8.906–50.008
Rather disagree	111	18 5.250	2.217–12.431	32 8.296	3.833–17.958
Partly	271	18 2.150	0.919–5031	45 4.779	2.290–9.972
Rather agree	433	14 1.047	0.433–2.529	26 1.728	0.797–3.745
**All**	**1104**	**82**		**134**	

* AIC = 66.316 BIC = 118.170; Reference category: “Agree” (unadjusted).

**Table 9:** Generalized linear models for the utilization of certain media platforms/channels associated with the perceived knowledgeability regarding COVID-19 vaccines.

Utilization of Certain Media Platforms/Channels and Perceived Knowledgeability *	Perceived Knowledgeability		
**“What are the most common information platforms you turn to for information on vaccines?”**	***n***	**OR**	**95% CI**
Public television channels (e.g., ARD, ZDF, Bayerischer Rundfunk)—“no”	950	1.012	0.861–1.191
Private TV channels (e.g., ProSieben, RTL) – “no”	2355	1.214	0.916–1.609
Daily newspapers (print or online)—“no”	1418	0.863	0.740–1.007
Online media (e.g., other websites)—“no”	1087	1,150	0.985–1.343
Radio—“no”	1981	1.027	0.856–1.231
Social networks (e.g., Facebook, Twitter)—“no”	2312	1.011	0.784–1.302
Podcasts—“no”	2267	1.011	0.802–1.276
Personal conversations with other people—“no”	1363	1.184	1.006–1.392
I do not seek specific information about vaccinations—“no”	2356	1.352	1.005–1.820
Utilization of certain media platforms/channels and COVID-19 vaccination intent **			
“What are the most common information channels you turn to for information on vaccines?” **	***n***	**OR**	**95% CI**
Scientific sources, e.g., peer-reviewed articles, reports of clinical trials—“no”	1306	1.024	0.873–1.201
Information from state or federal authorities (e.g., Federal Center for Health Education, Paul Ehrlich Institute or Robert Koch Institute)—“no”	826	0.772	0.650–0.917
Information from international organizations, e.g., World Health Organization—“no”	1846	1.099	0.925–1.305
Personal conversation with the (vaccinating) doctor or a medical professional (incl. the vaccinating healthcare professionals at the hospital’s vaccination centre)—”—“no”	2464	0.835	0.708–0.986
Information from health insurance companies—“no”	2282	0.926	0.620–1.382
Information from the local health department—“no”	2282	0.927	0.729–1.179
Information from pharmaceutical companies—“no”	2374	0.917	0.688–1.222
Information events, e.g., meetings with experts—“no”	2237	0.936	0.750–1.167
Personal conversations with family members, friends or acquaintances, colleagues—“no”	1663	1.233	1.044–1.457
I do not seek specific information channels to inform myself about vaccinations—“no”	2417	1.402	0.975–2.017
**All**	**2555**		

* Pearson’s Chi = 0.981 (GLM); ** “leftover”; Multiple choice questions; Reference category in each item is the answer “yes” to utilizing the given channel or platform.

**Table 10:** Multinomial logistic regression models for the utilization of certain media platforms/channels associated with the intent to receive a COVID-19 vaccine.

Utilisation of Certain Media Platforms/Channels Correlates with the Intent to Receive a COVID-19 Vaccine *	Yes (ref.)	No		Maybe	
**“What are the most common information platforms you turn to for information on vaccines?”**	***n***	***n*** **RR**	**95% CI**	***n*** **RR**	**95% CI**
Public television channels (e.g., ARD, ZDF, Bayerischer Rundfunk)—“no”	350	57 3.253	1.838–5.754	54 1.131	0.737–1.736
Private TV channels (e.g., ProSieben, RTL)—“no”	1008	73 0.619	0.266–1.442	124 (1.511; 0.267)	0.728–3.136
Daily newspapers (print or online)—“no”	596	61 1.811	0.999–3.283	97 2.282	1.482–3.514
Online media (e.g., other websites)—“no”	495	33 1.161	0.651–2.070	57 0.992	0.653–1.505
Radio—“no”	830	71 1.461	0.710–3.004	104 1.127	0.708–1.794
Social networks (e.g., Facebook, Twitter)—“no”	1004	60 0.308	0.166–0.571	123 (1.251; 0.520)	0.632–2.479
Podcasts—“no”	970	72 1.233	0.568–2.674	129 2.986	1.176–7.585
Personal conversations with other people—“no”	636	40 0.717	0.411–1.251	54 (0.516; 0.003)	0.335–0.794
I do not seek specific information about vaccinations—“no”	1027	64 0.591	0.275–1.270	115 0.683	0.442–1.708
“What are the most common information channels you turn to for information on vaccines?” **	**Yes (ref.)**	**No**		**Maybe**	
	***n***	***n*** **RR**	**95% CI**	***n*** **RR**	**95% CI**
Scientific sources, e.g., peer-reviewed articles, reports of clinical trials—“no”	627	37 0.526	0.295–0.936	85 1.045	0.688–1.587
Information from state or federal authorities (e.g., Federal Center for Health Education, Paul Ehrlich Institute or Robert Koch Institute)—“no”	355	55 3.434	1.926–6.123	60 1.339	0.862–2.079
Information from international organizations, eg. World Health Organization—“no”	798	58 0.507	0.275–0.935	97 0.685	0.432–1.087
Personal conversation with the (vaccinating) doctor or a medical professional (incl. the vaccinating healthcare professionals at the hospital’s vaccination centre)—“no”	814	65 1.156	0.618–2.162	104 1.403	0.878–2.241
Information from health insurance companies—“no”	1065	79 0.752	0.193–2.937	126 0.459	0.194–1.088
Information from the local health department—“no”	982	76 1.791	0.666–4.822	119 0.937	0.508–1.728
Information from pharmaceutical companies—“no”	1043	71 0.413	0.184–0.928	129 1.241	0.469–3.283
Information events, e.g., meetings with experts—“no”	982	68 0.583	0.292–1.163	123 1.199	0.608–2.364
Personal conversations with family members, friends or acquaintances, colleagues—“no”	742	46 0.598	0.346–1.034	64 0.448	0.293–0.686
I do not seek specific information channels to inform myself about vaccinations—“no”	1046	68 0.372	0.151–0.919	116 0.334	0.158–0.707
All	1104	82			134

* AIC = 1134.876 BIC = 1331.92; ** “leftover”; Reference category in each item is the answer “yes” to utilizing the given channel or platform (unadjusted).

## Data Availability

The data presented in this study are available on request from the corresponding author. The data are not publicly available due to the data protection policy of the LMU University Hospital.

## Supplementary Materials

The following supporting information can be downloaded at: https://www.mdpi.com/article/10.3390/vaccines10081231/s1, Supplements A and B can be found at the end of the manuscript.
