# Supplementary material for: COVID-19 Vaccination Intent, Barriers and Facilitators in Healthcare Workers: Insights from a Cross-Sectional Study on 2500 Employees at LMU University Hospital in Munich, Germany
Source: Vaccines (Basel). 2022 Jul 31;10(8):1231. doi: 10.3390/vaccines10081231 (PMC9412572; doi:10.3390/vaccines10081231)
Supplement: Supplementary file 1 [file vaccines-10-01231-s001.zip › Supplement_A.pdf]

vaccines

Article

The following tables provide further insights into the data collected through the implemented questionnaire. The data refers to the utilization of information sources within the LMU University hospital.

Table S1. Engagement with internal communication channels and platforms at LMU University Hospital

How often do you use the following information sources at LMU University Hospital?	Never		Rarely		Occasionally		Often		Always		Does not apply to me	
	N	%	N	%	N	%	N	%	N	%	N	%
Intranet (incl. Magazine “wir!online”)	131	5.1	330	12.9	623	24.4	870	34.1	574	22.5	27	1.1
IT-Portal (for use outside of the campus)	462	18.1	511	20.0	618	24.2	589	23.1	327	12.8	48	1.9
COVID-19 newsletter	58	2.3	131	5.1	292	11.4	746	29.2	1299	50.8	29	1.1
Print media (magazine “Klinikum aktuell”, posters, flyers)	642	25.1	837	32.8	606	23.7	311	12.2	117	4.6	42	1.6
Information from my manager	243	9.5	447	17.5	480	18.8	585	22.9	682	26.7	118	4.6
The hospital’s social media channels	1767	69.2	383	15.0	187	7.3	90	3.5	36	1.4	92	3.6
Information events for all of the hospital’s employees	845	33.1	620	24.3	508	19.9	309	12.1	190	7.4	83	3.2
Information events at my own department/clinic	521	20.4	432	16.9	411	16.1	492	19.3	453	17.7	246	9.6
Other mailings with employee information	92	3.6	182	7.1	357	14.0	690	27.0	1177	46.1	57	2.2
Scientific sources of the hospital/university	302	11.8	466	18.2	685	26.8	721	28.2	309	12.1	72	2.8
Direct contact with the vaccination center	1782	69.7	401	15.7	115	4.5	45	1.8	33	1.3	179	7.0

**Think about the information about the vaccination / vaccination center at the LMU Klinikum: To what extent do you agree with the following statements?**	**Disagree**		**Rather disagree**		**Partly**		**Rather agree**		**Agree**	
	**N**	**%**	**N**	**%**	**N**	**%**	**N**	**%**	**N**	**%**
In general, the LMU University Hospital has provided sufficient information regarding the in-house vaccination campaign.	146	5.7	273	10.7	671	26.3	873	34.2	592	23.2

The LMU University Hospital has provided sufficient information regarding the in-house vaccination process.	167	6.5	320	12.5	593	23.2	794	31.1	681	26.7
The LMU University Hospital has provided sufficient information regarding the utilized COVID-19 vaccines.	244	9.5	501	19.6	643	25.2	671	26.3	496	19.4
I generally felt well informed about the vaccination campaign at LMU University Hospital.	180	7.0	360	14.1	607	23.8	812	31.8	596	23.3
The information of the LMU University Hospital regarding the vaccination campaign are factual, objective and reliable.	85	3.3	140	5.5	422	16.5	1051	41.1	857	33.5
The information of the LMU University Hospital regarding the vaccination campaign easily understandable.	80	3.1	103	4.0	335	13.1	977	38.2	1060	41.5
The information of the LMU University Hospital regarding the vaccination campaign is up to date.	89	3.5	141	5.5	514	20.1	1018	39.8	793	31.0
It is important for me that information materials of LMU University Hospital quote relevant experts.	168	6.6	389	15.2	607	23.8	737	28.8	654	25.6
It is important for me to have a direct contact person to discuss my questions with.	186	7.3	347	13.6	454	17.8	765	29.9	803	31.4
I would have appreciated information from the LMU University Hospital in a different language besides German.*	1973	77.2	234	9.2	125	4.9	96	3.8	127	5.0

*Participants had the opportunity to suggest further languages in which the information should be provided. The following list represents the suggestions with five or more mentions (multiple suggestions were possible): All languages spoken by the hospital's employees n=245 (57,6%), English n=39 (9.2%), Polish n=31 (7.3%), Other languages (e.g. Bulgarian, Romanian, Chinese) n= 29 (6.8%), Russian n=19 (4.5%), Arabic n=14 (3.3%), Spanish n=14 (3.3%), French n=11 (2.6%), Serbo-Croatian n=8 (1.9%), Italian n=8 (1.9%), Turkish n=7 (1.6%)

Table S2. Utilization of the internal communication platforms at LMU University Hospital

**Which information/communication sources are you missing?**	**N**	**%**
A mobile application for employees	429	16.8
A comment function on the intranet page posts of the hospital	365	14.3
A hotline for questions regarding the COVID-19 vaccination	653	25.6
A group chat in a messenger app (e.g. WhatsApp)	184	7.2
No further sources or channels needed	1436	56.2
**Did you participate in the information event on the topic of vaccines organized on 15.12.2020?**	**N**	**%**
Yes	404	15.8
No	1273	49.8
I did not participate in the event, but I watched the video recording afterwards	469	18.4
I am not interested in such events	84	3.3
I do not remember	163	6.4
No answer	162	6.3
