# Supplementary material for: COVID-19 Vaccination Intent, Barriers and Facilitators in Healthcare Workers: Insights from a Cross-Sectional Study on 2500 Employees at LMU University Hospital in Munich, Germany
Source: Vaccines (Basel). 2022 Jul 31;10(8):1231. doi: 10.3390/vaccines10081231 (PMC9412572; doi:10.3390/vaccines10081231)
Supplement: Supplementary file 1 [file vaccines-10-01231-s001.zip › Supplement_B.pdf]

vaccines

Article

The following tables present the tested models that were not chosen for interpretation due to their poor model fit compared to the other model(s) chosen for interpretation.

Table S3 (adjusted). Multinomial logistic regression of attitudes towards vaccinations associated with intent to receive a COVID-19 vaccine

	Vaccination intent				
**"I think it's important that everyone receives the recommended vaccinations."***	**Yes (ref.)**	**No**		**Maybe**	
	**N**	**N<br>RR**	**95% CI**	**N<br>(RR**	**95% CI**
Disagree / rather disagree	13	65<br>649.302.	256.600 – 1642.999	32<br>56.369.	26.898 –<br>118.129
Partly	32	7<br>25.922.	9.026 - 74.451	50<br>33.607.	19.405 –<br>58.206
**„When you hear a negative comment about vaccine(s), do you:….."****					
"Ask for the opinion(s) of those in your in your private environment" - no	862	60<br>.736.	.412 - 1.1312	89<br>.203.	.327 -<br>.7775
"Get the opinion of a doctor or healthcare professional" - no	799	65<br>1.890.	1.017 - 3.513	100<br>1.394.	.889 –<br>2.186
"Check the correctness of the statements through media reports" – no	328	30<br>1.579.	.804 - 3.100	43<br>.965.	.580 -<br>1.604
"I do not (often) deal with negative comments" – no	865	73<br>2,633	1.121 - 6.182	111<br>1,141	.652 -<br>1.996
"No answer" - no	1038	69<br>.632	.247 - 1.613	120<br>.508	,229 -<br>1.127
„I engage with the person expressing the negative comment" – no***	1097	82		1134	
"Did you get vaccinated against influenza in 2020/21 season?"****	**Yes (ref.)**	**No**		**Maybe**	
	N	N<br>RR	95% CI	N<br>(RR; p-value)	95% CI
"Yes"	665	13<br>.125	.067 - .232	29<br>.186	.120<br>-.287
"No" (ref.)	439	69		105	
All (not yet vaccinated)	**1104**	**82**		**134**	

*AIC =398.414 BIC = 564.346, adjusted for age, education; Reference category. Agree / rather agree; **Multiple choice question, AIC = 853.136 BIC = 1050,180, adjusted for age, education; Reference category in each item is the answer „yes" to executing the given action; ***Too few cases to allow for analysis; ****AIC = 410.415 BIC = 565.977, adjusted for age;

Table S4 (unadjusted). Binomial logistic regression of attitudes towards vaccinations associated with negative COVID-19 vaccination status

**"To what extent do you agree with the following statement? "**	**Vaccination status (not vaccinated)**		
**"I find it important for everyone to receive the recommended vaccinations"**	**N**	**OR**	**95% CI**
Disagree / rather disagree	110	.158	.095 – .261
Partly	89	.574	.404 - .814
**„When you hear a negative comment about vaccine(s), do you:….."****			
	**N**	**OR**	**95% CI**
"Ask for the opinion(s) of those in your private environment" – no	1011	1.139	.937 - 1.385
"Get the opinion of a doctor or healthcare professional" – no	964	.935	.778 - 1.124
"Check the correctness of the statements through media reports" – no	401	1.049	.851 - 1.293
"I do not (often) deal with negative comments" – no	1049	.916	.732 - 1.146
"No answer" – no	1227	2.012	1.329 - 3.048
„I engage with the person expressing the negative comment" – no****	1313	-	-
All	**1320**		

* Cox & Snell R-Quadrat= .031 Nagelkerkes R-Quadrat =.041; Reference category; Agree / rather agree; **Multiple choice question, Cox & Snell R-Quadrat = .007 Nagelkerkes R-Quadrat =009; Reference category in each item is the answer „yes" to executing the given action (***too few cases to allow for analysis)

Table S5 (adjusted). Binomial logistic regression of negative COVID-19 vaccination status associated with the attitudes towards other implemented nonpharmaceutical measures

**"In general regard to the COVID-19 vaccination campaign, it is important for me…"***	**Vaccination status (not vaccinated)**		
**"…that the current measures at LMU University Hospital (e.g. mask mandate) remain valid until the end of 2021"**	**N**	**OR**	**95% CI**
Disagree	90	.957	.610 - 1.500
Rather disagree	85	.911	.597 - 1.390
Partly agree	235	1.152	.878 – 1.511
Rather agree	347	1.432	1.147 - 1.787
**"…that the current measures at LMU University Hospital (e.g. mask mandate) remain valid in 2022 as well"**	**N**	**OR**	**95% CI**
Disagree	210	.711	.496 - 1.019
Rather disagree	216	.660	.481 - .905
Partly agree	439	.723	.560 - .933
Rather agree	228	.756	.577 - .992

**"...that testing at the LMU University Hospital should remain broadly available regardless of the vaccination campaign"**	**N**	**OR**	**95% CI**
Disagree	34	.330	.157 - .693
Rather disagree	23	.646	.326 - 1.282
Partly agree	76	.753	.521 - 1.089
Rather agree	361	.792	.652 - .962
All	**1320**		

*Cox & Snell R-Quadrat = ,016 Nagelkerkes R-Quadrat = .021; the distribution of answers allowed for testing without merging any categories; Reference category in each item is the answer „Agree";

Table S6 (adjusted). Multinomial logistic regression models with the Health Belief Model factors associated with intent to receive a COVID-19 vaccine

**Perceived susceptibility is a predictor for getting a COVID-19 vaccine***	**Vaccination intent**				
**α = 0,509 AIC = 992,897 BIC = 1241,795**	**Yes (ref.)**	**No**		**Maybe**	
**"How do you rate the following aspects from your personal point of view?"**	**N**	**N<br>RR**	**95% CI**	**N<br>RR**	**95% CI**
**"In regard to the spread of COVID-19 the likelihood that I myself be will infected is..."**					
Very low / Low	337	51<br>..806.	.292 - 2.228	58<br>1..735.	.767 -<br>3.925
Medium	571	21<br>..505.	.186 - 1.365	62<br>1.045.	.497<br>2.1<br>99
**"Since the vaccination campaign started, I've been more afraid of getting infected in my private environment than before or I've been more afraid for my loved ones."**	**N**	**N<br>RR**	**95% CI**	**N<br>RR**	**95% CI**
Disagree / Rather disagree	892	76<br>.839.	.274 - 2.574	106<br>.717.	.320 -<br>1.604
Partly	152	2<br>.426.	.066 - 2.744	20<br>.831.	.321<br>2.1<br>48
**"Since the vaccination campaign started, I've been less afraid of getting infected in my private environment than before or I've been less more afraid for my loved ones."**	**N**	**N<br>RR**	**95% CI**	**N<br>RR**	**95% CI**
Disagree / Rather disagree	571	70<br>2.050.	.894 - 5.196	90<br>(1.895;<br>0.083)	.920 -<br>3.906
Partly	255	2<br>.263.	.050 - 1.398	31<br>1.655.	.758 -<br>3.615
**"Since the vaccination campaign started, I've been less afraid of getting infected in my professional environment than before."**	**N**	**N<br>RR**	**95% CI**	**N<br>RR**	**95% CI**
Disagree / Rather disagree	575	71<br>2.628.	.940 - 7.346	93<br>2.996.	1.387 -<br>6.472
Partly	248	3<br>.480.	.109 - 2.108	30<br>2.294.	1.029 -<br>5.114

**"Since the vaccination campaign started, I've been more afraid of getting infected in my professional environment than before."**	**N**	**N RR**	**95% CI**	**N (RR; p-value)**	**95% CI**
Disagree / Rather disagree	925	78 7.286.	2.239 - 23.718	109 2.234.	.899 - 5.554
Partly	124	2 1.478.	.458 - 4.764	18 2.057.	.889 - 4.759
**Perceived severity is a predictor for a getting a COVID-19 vaccine**	**Yes (ref.)**	**No**		**Maybe**	
**α = 0,817 AIC = 631,736 BIC = 818,409**					
**"How do you rate the following aspects from your personal point of view?"**	**N**	**N RR**	**95% CI**	**N RR**	**95% CI**
**"In regard to the spread of COVID-19 the probability of me getting sick from COVID-19 is..."**					
Very low / Low	370	60 2.361.	.859 - 6.492	59 2.323.	1.018 - 5.301
Medium	562	16 0.577.	.206 - 1.621	65 1.798.	.831 - 3.891
**"In regard to the spread of COVID-19 the probability of me getting seriously ill from COVID-19 is..."**	**N**	**N RR**	**95% CI**	**N RR**	**95% CI**
Very low / Low	654	72 7.206.	.845 - 61.482	91 1.652 .	.610 - 4.472
Medium	342	9 3.197.	.366 - 27.898	37 1.357.	.507 3.6 30
**Perceived benefits are a predictor for a getting a COVID-19 vaccine**	**Yes (ref.)**	**No**		**Maybe**	
**AIC = 445,166 BIC = 611,098**					
**"I am completely convinced of the effectiveness of the COVID-19 vaccines"**	**N**	**N RR**	**95% CI**	**N RR**	**95% CI**
Disagree / Rather disagree	17	63 799.199.	290.349 - 2199.823	46 98.278.	48.215 - 200.322
Partly	170	12 11.704.	4.391 - 31.202	54 9.342.	5.758 15. 157
**Perceived barriers are a predictor for a getting a COVID-19 vaccine**	**Yes (ref.)**	**No**		**Maybe**	
**α = 0,845 AIC = 561,019 BIC = 747,693**	**N**	**N RR**	**95% CI**	**N RR**	**95% CI**
**"I am completely convinced of the safety of the COVID-19 vaccines"**					
Disagree / Rather disagree	33	71 169..818.	40.369 - 714.364	59 23.034.	10.456 - 50.745
Partly	215	8 6.428	1.432 - 28.859	57 6.038	3.111 - 11.717
"I have no concerns regarding the COVID-19 vaccines"	**N**	**N RR**	**95% CI**	**N RR**	**95% CI**

Disagree / Rather disagree	93	73<br>9.772	2.747 -<br>34.766	81<br>7.721	3.794 -<br>15.712
Partly	215	5<br>1.696	.370 - 7.772	36<br>2.782	1.366 -<br>5.655
All	**1104**	**82**			**134**

* Reference category in each item is the highest answer on the merged Likert scale („Rather agree / Agree" or „High / Very high")

Table S7 (unadjusted). Binomial logistic regression models with the Health Belief Model factors associated with intent to receive a COVID-19 vaccine

**Perceived susceptibility is a predictor for getting a COVID-19 vaccine*°**	**Vaccination status (not vaccinated)**		
**"How do you rate the following aspects from your personal point of view?"**	**N**	**OR**	**95% CI**
**"In regard to the spread of COVID-19 the likelihood that I myself be will infected is..."**			
Very low / Low	446	.484	.337 - .696
Medium	654	.708	.520 - .963
**"Since the vaccination campaign started, I've been more afraid of getting infected in my private environment than before or I've been more afraid for my loved ones."**			
Disagree / Rather disagree	1074	1.365	.880 - 2.116
Partly	174	.915	.543 - 1.540
"Since the vaccination campaign started, I've been less afraid of getting infected in my private environment than before or I've been less afraid for my loved ones."			
Disagree / Rather disagree	731	.370	.286 - .480
Partly	288	.580	.443 - .758
**"Since the vaccination campaign started, I've been less afraid of getting infected in my professional environment than before."**			
Disagree / Rather disagree	739	.219	.169 - .283
Partly	281	.489	.379 - .631
**"Since the vaccination campaign started, I've been more afraid of getting infected in my professional environment than before."**			
Disagree / Rather disagree	489	2.079	1.415 - 3.053
Partly	643	1.122	.796 - 1.582
**Perceived severity is a predictor for a getting a COVID-19 vaccine****			
**"In regard to the spread of COVID-19 the probability of me getting sick from COVID-19 is..."**	**N**	**OR**	**95% CI**
Very low / Low	489	1.158	.856 - 1.565
Medium	643	0.823	.624 - 1.087
**"In regard to the spread of COVID-19 the probability of me getting seriously ill from COVID-19 is..."**			
Very low / Low	817	1.388	.973 - 1.980

Medium	388	0.954	.668 - 1.364
**Perceived benefits are a predictor for a getting a COVID-19 vaccine*** **			
**"I am completely convinced of the effectiveness of the COVID-19 vaccines"**	**N**	**OR**	**95% CI**
Disagree / Rather disagree	126	.078	.042 - .145
Partly	236	.558	.447 -. 698
**"I am completely convinced of the effectiveness of the COVID-19 vaccines"**	**N**	**OR**	**95% CI**
Disagree / Rather disagree	126	.078	.042 - .145
Partly	236	.558	.447 - .698
**Perceived barriers are a predictor for a getting a COVID-19 vaccine********	**N**	**OR**	**95% CI**
**"I am completely convinced of the safety of the COVID-19 vaccines"**			
Disagree / Rather disagree	163	.216	.129 - .361
Partly	280	.701	.547 - .899
**"I have no concerns regarding the COVID-vaccines"**			
Disagree / Rather disagree	247	.439	.332 - .661
Partly	256	.740	.577 - .948
All	**1320**		

° Reference category in each item is the highest answer on the merged Likert scale („Rather agree / Agree" or „High / Very high")
* Cox & Snell R-Quadrat = .218 Nagelkerkes R-Quadrat = .290**Cox & Snell R-Quadrat= .019 Nagelkerkes R-Quadrat= .025 *** Cox & Snell R-Quadrat= .052 Nagelkerkes R-Quadrat = .070 **** Cox & Snell R-Quadrat= .065 Nagelkerkes R-Quadrat = .086

Table S8 (adjusted). Multinomial logistic regression models for the perceived knowledgeability associated with intent to receive a COVID-19 vaccine

Perceived knowledgability is a predictor of intent to receive a COVID-19 vaccine*	Yes (ref.)	No		Maybe	
"I generally felt well informed about COVID-19 vaccines and their safety"	N	N<br>RR	95% CI	N<br>RR	95% CI
Disagree	30	24<br>19.961	8.079 - 49.321	22<br>19.182	7.980 -<br>46.105
Rather disagree	111	18<br>4.407	1.820 - 10.668	32<br>(8.142	3.715 -<br>17.845
Partly	271	18<br>1.194	.823 - 4.593	45<br>4.461	2.124 -<br>9.369
Rather agree	433	14<br>0.948	.389 - 2.308	26<br>1.672	.768 -<br>3.639

All	**1104**	**82**	**134**

* AIC = 663,511 BIC = 850,185; Reference category: "Agree"

Table S9 (unadjusted). Generalized linear models for the utilization of certain media platforms/channels associated with the perceived knowledgeability regarding COVID-19 vaccines

Utilization of certain media platforms/channels and perceived knowledgeability*	Perceived knowledgeability		
"What are the most common information platforms you turn to for information on vaccines?"	N	OR	95% CI
Public television channels (e.g. ARD, ZDF, Bayerischer Rundfunk) – „no"	950	1.025	.875 - 1.203
Private TV channels (e.g. ProSieben, RTL) - „no"	2355	1.302	.987 - 1.718
Daily newspapers (print or online) - „no"	1418	.844	.726 - .982
Online media (e.g. other websites) - „no"	1087	1.138	.975 - 1.327
Radio - „no"	1981	1.010	.846 - 1.207
Social networks (e.g. Facebook, Twitter) - „no"	2312	1.174	.916 - 1.505
Podcasts - „no"	2267	.959	.762 - 1.206
Personal conversations with other people - „no"	1363	1.174	1.000 - 1.378
I do not seek specific information about vaccinations - „no"	2356	1.303	.969 - 1.752
Utilization of certain media platforms/channels and COVID-19 vaccination intent**			
"What are the most common information channels you turn to for information on vaccines?"**	N	OR	95% CI
Scientific sources, e.g. peer-reviewed articles, reports of clinical trials – „no"	1306	.903	.774 - 1.054
Information from state or federal authorities (e.g. Federal Center for Health Education, Paul Ehrlich Institute or Robert Koch Institute) – „no"	826	.769	.648 - .912
Information from international organizations, e.g. World Health Organization – „no"	1846	1.149	.970 - 1.360
Personal conversation with the (vaccinating) doctor or a medical professional (incl. the vaccinating healthcare professionals at the hospital's vaccination center) – „no"	2464	0.879	.740 - 1.026
Information from health insurance companies – „no"	2282	0.967	.650 - 1.439
Information from the local health department – „no"	2282	0.949	.749 - 1.203
Information from pharmaceutical companies – „no"	2374	0.868	.652 - 1.156
Information events, e.g. meetings with experts – „no"	2237	0.908	.730 - 1.131
Personal conversations with family members, friends or acquaintances, colleagues – „no"	1663	1.324	1.124 - 1.560
I do not seek specific information channels to inform myself about vaccinations – „no"	2417	1.641	1.144 - 2.355
All	**2555**		

*Pearson Chi = 0,961 (GLM); Multiple choice questions; Reference category in each item is the answer „yes" to utilizing the given channel or platform

Table S10 (adjusted). Multinomial logistic regression models for the utilization of certain media platforms/channels associated with the intent to receive a COVID-19 vaccine

Utilisation of certain media platforms/channels correlates with the intent to receive a COVID-19 vaccine *	Yes (ref.)	No		Maybe	
"What are the most common information platforms you turn to for information on vaccines?"	N	N<br>RR	95% CI	N<br>RR	95% CI
Public television channels (e.g. ARD, ZDF, Bayerischer Rundfunk) – „no“	350	57<br>3.328	1.826 - 6.066	54<br>1.107	.711 - 1.724
Private TV channels (e.g. ProSieben, RTL) - „no“	1008	73<br>.654	.267 - 1.599	124<br>1.164	.786 - 3.525
Daily newspapers (print or online) - „no“	596	61<br>1.693	.897 3.193	97<br>2.364	1.509 - 3.704
Online media (e.g. other websites) - „no“	495	33<br>1.119	.613 - 2.045	57<br>1.025	.670 - 1.568
Radio - „no“	830	71<br>1.613	.761 - 3.416	104<br>1.164	.719 - 1.885
Social networks (e.g. Facebook, Twitter) - „no“	1004	60<br>0.319	.166 - .615	123<br>1.528	.621 - 2.548
Podcasts - „no“	970	72<br>1.324	.577 - 3.039	129<br>3.075	1.185 - 7.982
Personal conversations with other people - „no“	636	40<br>.851	.469 - 1.546	54<br>0.512	.329 - .796
I do not seek specific information about vaccinations - „no“	1027	64<br>.554	.252 - 1.216	115<br>.816	.411 - 1.620
"What are the most common information channels you turn to for information on vaccines?"**	**Yes (ref.)**	**No**		**Maybe**	
	N	N<br>RR	**95% CI**	N<br>RR	**95% CI**
Scientific sources, e.g. peer-reviewed articles, reports of clinical trials – „no“	627	37<br>.459	.251 -.842	85<br>.947	.615 - 1.459
Information from state or federal authorities (e.g. Federal Center for Health Education, Paul Ehrlich Institute or Robert Koch Institute) – „no“	355	55<br>3.745	2.043 - 6.864	60<br>1,362	.867 - 2.139
Information from international organizations, eg. World Health Organization – „no“	798	58<br>.470	.249 - .886	97<br>.649	.404 - 1.043
Personal conversation with the (vaccinating) doctor or or a medical professional (incl. the vaccinating healthcare professionals at the hospital's vaccination center) – „no“	814	65<br>1.129	.674 - 2.473	104<br>1,450	.899 - 2.337
Information from health insurance companies – „no“	1065	79<br>.966	.224 - 4.172	126<br>.480	.201 - 1.148
Information from the local health department – „no“	982	76<br>1.667	.604 - 4.597	119<br>.896	.482 - 1.665

Information from pharmaceutical companies – „no“	1043	71<br>.431	.184 - 1.008	129<br>1,195	.464 - 3.357
Information events, e.g. meetings with experts – „no“	982	68<br>.607	.292 - 1.262	123<br>1,195	.600 - 2.379
Personal conversations with family members, friends or acquaintances, colleagues – „no“	742	46<br>.589	.330 - 1.053	64<br>.438	.284 - .675
I do not seek specific information channels to inform myself about vaccinations – „no“	1046	68<br>.421	.165 - 1.071	116<br>.334	.155 - .723
All	**1104**	**82**			**134**

* AIC = 1321.048 BIC = 1663.284; Reference category in each item is the answer „yes“ to utilizing the given channel or platform
